# PPR596 Is Required for *nad2* mRNA Splicing and Complex I Biogenesis in Mitochondria of *Arabidopsis thaliana*

**DOI:** 10.1111/ppl.70507

**Published:** 2025-09-09

**Authors:** Leonora Peters, Vera C. Wagner, Theresa Schoeller, Sarlita Dwiani, Mareike Schallenberg‐Rüdinger, Etienne H. Meyer, Kristina Kühn

**Affiliations:** ^1^ Department of Plant Physiology, Institute of Biology Martin‐Luther‐Universität Halle‐Wittenberg Halle (Saale) Germany; ^2^ Institut für Zelluläre Und Molekulare Botanik, Abteilung Molekulare Evolution Universität Bonn Bonn Germany

**Keywords:** plant mitochondria, PPR protein, splicing

## Abstract

Several genes in the mitochondria of angiosperms are interrupted by introns, and their posttranscriptional excision involves numerous nucleus‐encoded auxiliary factors. Most of these factors are of eukaryotic origin, among them members of the pentatricopeptide‐repeat (PPR) family of RNA‐binding proteins. This family divides into the PLS and P classes, with PLS‐class proteins typically participating in C‐to‐U mRNA editing and P‐class members contributing to transcript stabilization and intron splicing. The P‐class protein PPR596 was previously described to be involved in mitochondrial RNA editing, with the *ppr596* mutant showing moderately elevated editing of a specific, partially edited site within the *rps3* mRNA. PPR596 disruption led to a substantial delay in plant development. Because the moderate change in RNA editing in the *ppr596* mutant is unlikely to be the cause of its severe developmental retardation, we re‐investigated mitochondrial gene expression and found that PPR596 is specifically required for the efficient excision of the third intron from the *nad2* pre‐mRNA. Insufficient splicing of this intron in *ppr596* impairs respiratory‐chain complex I biogenesis at the step of the insertion of the Nad2 subunit, thus perturbing plant development.

## 1 Introduction

Plant mitochondria perform crucial functions in energy metabolism, and core components of the mitochondrial oxidative phosphorylation (OXPHOS) system are encoded by the mitochondrial genome. Mitochondrial genomes of land plants differ from those of animals in that they contain substantial non‐coding regions, including introns (Gualberto and Newton 2017), and that they require an extraordinarily large number of nucleus‐encoded proteins for the expression of mitochondrial genes (Small et al. 2023). Most of these proteins participate in RNA maturation processes, which include C‐to‐U RNA editing by cytidine deamination, intron splicing, and transcript 5′ and 3′ end maturation. The question of why gene expression in plant mitochondria has evolved to be substantially more complex than in their bacterial ancestors and other eukaryotes has been addressed by different hypotheses (Best et al. 2020; Small et al. 2023).

Introns found in plant mitochondria and plastids originate from mobile ribozymes that have invaded these genomes during evolution (Bonen 2012; Lambowitz and Zimmerly 2011). Based on their structure and biochemistry of splicing, organellar introns have been assigned to two different groups, I and II (Bonen 2012). Excepting the curious case of a group‐I intron with sporadic occurrence across angiosperms and probably spread via horizontal transfer (Cho et al. 1998), all introns found in higher plant mitochondrial genomes are identified as group‐II introns (Bonen 2008). The majority of these introns are located in *nad* genes encoding subunits of respiratory‐chain complex I, with the *nad1*, *nad2*, and *nad5* mRNAs requiring both *cis*‐ and *trans*‐splicing for their maturation (Bonen 2008).

Group‐II introns have a distinctive secondary structure with six domains (dI‐dVI) extending from a central hub; tertiary interactions between these domains mediate productive intron folding (reviewed in Lambowitz and Zimmerly (2011), Michel and Ferat (1995), and Bonen and Vogel (2001)). dV and dVI are particularly important for the biochemistry of splicing, the conserved dV forming part of the catalytic core of the ribozyme and dVI containing an unpaired adenosine which functions as the attacking nucleophile during the first step of splicing. Canonical group II introns splice via two consecutive transesterifications, resulting in the formation of an excised intron lariat (Lambowitz and Zimmerly 2011). An alternative group II intron splicing mechanism has been observed, whereby the first step of splicing occurs by hydrolysis and introns are released as linear or circular molecules; examples also exist in angiosperm mitochondria (Dalby and Bonen 2013; Gualberto et al. 2015; Li‐Pook‐Than and Bonen 2006).

The excision of mitochondrial group‐II introns from pre‐mRNAs requires the participation of intron maturases, RNA helicases, and diverse other protein cofactors (see Edris et al. 2024, Small et al. 2023 for recent overviews). Apart from the intron maturase MatR encoded within the fourth intron of the mitochondrial *nad1* gene (Schmitz‐Linneweber et al. 2015), all components of the mitochondrial splicing machinery are encoded in the nucleus. They have mostly been identified by homology to intron‐encoded maturases or previously characterized chloroplast splice factors and through analyses of loss‐of‐function mutants in Arabidopsis and maize. The molecular function of mitochondrial maturases in transcript splicing remains to be defined; they deviate from ancestral bacterial intron maturases in that they each aid the splicing of multiple introns (Schmitz‐Linneweber et al. 2015). Other factors promoting the splicing of several mitochondrial introns are RNA helicases (Mizrahi and Ostersetzer‐Biran 2024) and CRM‐domain proteins (Lee et al. 2019; Lin et al. 2022; Zmudjak et al. 2013). Both are considered to contribute to productive intron folding: RNA helicases by resolving incorrectly folded RNA structures, and CRM‐domain proteins by binding to RNA and acting as RNA chaperones (Barkan et al. 2007; Lee et al. 2014; Small et al. 2023). Further factors required for the splicing of more than one mitochondrial intron are the PORR‐domain RNA‐binding proteins RPD1 and WTF9 (Colas Des Francs‐Small et al. 2012; Edris et al. 2024; Wang et al. 2024) and the RanBP2‐type zinc finger protein OZ2 (Bentolila et al. 2021). A number of mitochondrial splice factors have been identified that belong to protein families not normally associated with transcript splicing; those include the RCC1‐like protein RUG3, the RAD52‐like protein ODB1, and the ribosomal uL18 family protein uL18‐L1 (Gualberto et al. 2015; Wang et al. 2020; Kühn et al. 2011). Most known factors contributing to intron excision in plant mitochondria, however, are helical‐repeat modular proteins of the pentatricopeptide repeat (PPR) family (Wang and Tan 2025; Edris et al. 2024 and references therein). The mode by which they promote splicing is largely unresolved.

PPR proteins usually bind to single‐stranded RNA, with each PPR motif interacting with a single RNA nucleotide via two amino acids that define the specificity of this interaction (Barkan et al. 2012; Barkan and Small 2014; Shen et al. 2016). PPR proteins sort into two different classes, according to the types of PPR motifs they are composed of (Barkan and Small 2014). P‐class PPR proteins usually engage in tight, passive, and highly specific binding to noncoding RNA sequences and function in intron splicing and RNA stabilization (Small et al. 2023). PLS‐class PPR proteins can associate with more variable target sequences and typically function as site‐specificity factors in RNA editing (Barkan and Small 2014) and, for PLS‐class proteins with a DYW domain, as cytidine deaminases in the editing process (Oldenkott et al. 2019; Small et al. 2020). PLS‐class protein architecture is not incompatible with functions normally performed by P‐class proteins: In plastids of Arabidopsis, the PLS‐class proteins OTP70 and CRR2 are required for the splicing and stabilization, respectively, of highly specific mRNA targets (Chateigner‐Boutin et al. 2011; Hashimoto et al. 2003; Ruwe et al. 2019). On the other hand, an unusual P‐class PPR protein with seemingly many targets is PPR‐SMR1, which in maize mitochondria is required for the splicing of 15 or more mitochondrial introns (Chen et al. 2019). Another study has reported on the functioning of a mitochondrial P‐class PPR protein, PPR596, in RNA editing (Doniwa et al. 2010). PPR596 disruption in Arabidopsis was described to positively affect C‐to‐U editing of a specific, partially edited site within the *rps3* mRNA and led to a substantial delay in development in comparison with wild‐type plants.

Because the moderate change detected in RNA editing in the *ppr596* mutant is unlikely to be the cause of its severe developmental retardation, we re‐investigated mitochondrial gene expression and the accumulation of protein complexes of the OXPHOS system in *ppr596*. Very recent work indicated a role for this protein in the splicing of the mitochondrial *nad2* mRNA (Sayyed et al. 2024). The work presented here confirms the role of the mitochondrial PPR protein PPR596 in *nad2* mRNA splicing in Arabidopsis and shows that it is specifically required for the efficient excision of the third intron of *nad2*. Accordingly, *ppr596* fails to accumulate normal levels of respiratory‐chain complex I, for which *nad2* encodes a core subunit.

## 2 Materials and Methods

### 2.1 Plant Material and Culture Conditions

The *ppr596* mutant was isolated from T‐DNA insertion line SAIL_367_A06 (Sessions et al. 2002) obtained from the European Arabidopsis Stock Centre (https://arabidopsis.info/). Complemented lines Comp1 and Comp2 were generated as described under 2.2. Surface‐sterilized seeds were sown on agar plates containing 0.5× MS255 (Duchefa) and supplemented with 1% w/v sucrose, stratified at 4°C for 3 days in the dark, and subsequently exposed to a light intensity of 100 μmol quanta m^−2^ s^−1^ in a 16‐h photoperiod at 22°C in a growth chamber with LED illumination. For nucleic acid extractions from seedlings, seedlings were harvested at growth stage 1.02 according to Boyes et al. (2001). For the extraction of mitochondria, seedlings were transferred to soil at stage 1.02 and grown at 100 μmol quanta m^−2^ s^−1^ in a 16‐h photoperiod at 22°C in a growth chamber with LED illumination until reaching stage 5.10 (Boyes et al. 2001).

### 2.2 *ppr596* Mutant Complementation

A 3.7‐kb genomic fragment comprising the *PPR596* gene was PCR‐amplified with Phusion High‐Fidelity DNA Polymerase (Thermo Fisher Scientific Inc.) and primers listed in Table S1, and ligated into the pGEM‐T Easy vector (Promega GmbH). The resulting plasmid was cloned and partially digested with ApaI and NotI, and the 3.77 fragment obtained was ligated between the ApaI and NotI sites of a modified pORE‐E3 vector (Coutu et al. 2007) in which the resistance gene *PAT* had been replaced by *HPT*. The resulting vector was used to transform *Agrobacterium tumefaciens* GV3101, and the strain obtained was used for the transformation of *ppr596* plants by floral dip. T1 seedlings were screened on plates containing 20 μg/mL hygromycin. Two independent resistant lines were kept for further analysis and named Comp1 and Comp2.

### 2.3 DNA And RNA Isolation, Reverse Transcription, and PCR

Genomic DNA isolated as previously described (Edwards et al. 1991) was used for PCR‐based genotyping analysis with DreamTaq DNA Polymerase (Thermo Fisher Scientific Inc.; see Table S1 for primer information). Seedling RNA was isolated using TRIzol reagent (Thermo Fisher Scientific Inc.) according to the manufacturer's instructions. For all subsequent procedures except RNA gel blot hybridizations, RNA samples were depleted of contaminating genomic DNA by treatment with RQ1 RNase‐free DNase (Promega GmbH) and confirmed by PCR to be free of detectable amounts of DNA. To produce cDNA for quantitative RT‐PCR experiments and analyses of mRNA editing, 1 μg of RNA was reverse‐transcribed with RevertAid H Minus reverse transcriptase (Thermo Fisher Scientific Inc.) according to the manufacturer's protocol using random hexamers.

### 2.4 Quantitative RT‐PCR

Quantitative RT‐PCR (RT‐qPCR) for measuring mitochondrial transcripts was performed using SYBR Green I Master Mix (Roche Diagnostics GmbH) and primer pairs listed in Table S1 as previously described (Falcon De Longevialle et al. 2007) on a LightCycler 480 real‐time PCR system (Roche Diagnostics GmbH). The nuclear 18S rRNA (At3g41768) and *ACT* genes (At1g49240, At3g18780) were used for data normalization.

### 2.5 RNA Gel Blot Hybridization

2 μg total RNA extracted from Arabidopsis seedlings was resolved on 1.2% (w/v) agarose‐formaldehyde gels and transferred onto positively charged Nylon membranes (Roche Diagnostics GmbH). Digoxigenin‐labeled DNA probes were generated using the PCR DIG Probe Synthesis Kit (Roche Diagnostics GmbH) and primer pairs listed in Table S1. Hybridizations were performed at 50°C in DIG Easy Hyb solution (Roche Applied Science) according to the manufacturer's instructions; chemiluminescent detection of signals was done using Anti‐digoxigenin‐AP conjugates and CSPD reagent (Roche Applied Science) in a FUSION FX EDGE chemiluminescence detection system (Vilber Lourmat).

### 2.6 Analysis of Transcript Editing

cDNA fragments were PCR‐amplified from cDNA samples produced as detailed under 2.3 with primers listed in Table S1 and sent for Sanger sequencing at Microsynth Seqlab GmbH.

### 2.7 Prediction of PPR596 Binding Sites

PPR motifs and amino acids at the specificity‐defining fifth and last positions (Barkan et al. 2012) were identified using the PPR finder tool “PPR”, which is available at https://ppr.plantenergy.uwa.edu.au/ (Cheng et al. 2016; Gutmann et al. 2020). Nucleotide preferences for amino acid combinations of each PPR were taken from (Barkan et al. 2012) and (Yan et al. 2019). Prediction of putative PPR596 binding sites in the sequence comprising exon 3, intron 3, and exon 4 of *nad2* was performed using the Target Scan module of the Plant RNA‐Editing Prediction and Analysis computer tool (PREPACT, http://www.prepact.de/prepact‐main.php, (Lenz et al. 2018)).

### 2.8 Isolation of Mitochondria From Arabidopsis Plants

For the isolation of mitochondria from rosette leaf tissue, Arabidopsis plants were grown to reach growth stage 5.10 (Boyes et al. 2001) as detailed under 2.1. 20–40 g of aerial parts of plants were harvested and ground at 4°C in ~50 mL extraction buffer (0.3 M sucrose, 25 mM K_4_P_2_O_7_, 10 mM KH_2_PO_4_, 2 mM EDTA, 1% [w/v] PVP‐40, 1% [w/v] BSA, 5 mM cysteine, and 25 mM sodium ascorbate, pH 7.5) per 10 g of plant material using a mortar and a pestle. The homogenate was spun at 1700 *g* for 5 min and the supernatant was spun at 20,000 *g* for 10 min. The pellet was resuspended in wash buffer (0.3 M sucrose, 1 mM EGTA, and 10 mM MOPS/KOH, pH 7.2) using a paint brush and subjected to additional low‐ (2500 *g*, 10 min) and high‐speed (20,000 *g*, 10 min) centrifugations. The pellet was resuspended in a small volume (around 1 mL) of wash buffer and loaded on top of a Percoll step gradient (from bottom to top: 5 mL 50%, 25 mL 28% and 5 mL 18% Percoll in wash buffer, equivalent to ~20 g of plant material loaded per gradient). The gradient was centrifuged at 40,000 *g* for 45 min, and the mitochondrial fraction located at the interface between the 50% and 28% layers was collected, washed in wash buffer, and spun down at 20,000 *g* for 10 min. This wash of mitochondria was repeated twice, after which the protein concentration of mitochondrial samples was estimated using Rotiquant assay (Carl Roth GmbH). Mitochondria were kept at 4°C throughout the purification procedure. They were stored as a mitochondrial suspension in wash buffer at −80°C.

### 2.9 Blue‐Native PAGE and Immunodetection of Protein Complexes

For Blue‐Native (BN)‐PAGE, mitochondrial samples equaling 100 μg of protein were solubilized with 5% (w/v) digitonin, and solubilized protein complexes were separated on a BN gel according to (Eubel et al. 2005). Following migration, gels were Coomassie‐stained or subjected to in‐gel activity assaying (see 2.10), or protein complexes were transferred onto a PVDF membrane (Immobilon‐P, Millipore) in BN cathode buffer without Coomassie (50 mM Tricine, 15 mM Bis‐Tris, pH 7.0) using a wet‐blotting system. Membrane‐immobilized complexes were probed with antibodies against CA2 (Röhricht et al. 2023) using the procedure detailed in (Röhricht et al. 2023).

### 2.10 In‐Gel NADH Oxidase Activity Assay

The assay was performed according to (Zerbetto et al. 1997). The gel was washed 3 times for 5 min with deionized water and incubated in the reaction medium (0.14 mM NADH, 1.22 mM nitro blue tetrazolium, 0.1 M Tris–HCl, pH 7.4). When the dark‐blue stain was strong enough, the reaction was stopped by transferring the gel into deionized water.

## 3 Results and Discussion

### 3.1 PPR596 Is Required for *nad2* Transcript Splicing

**FIGURE 1:**
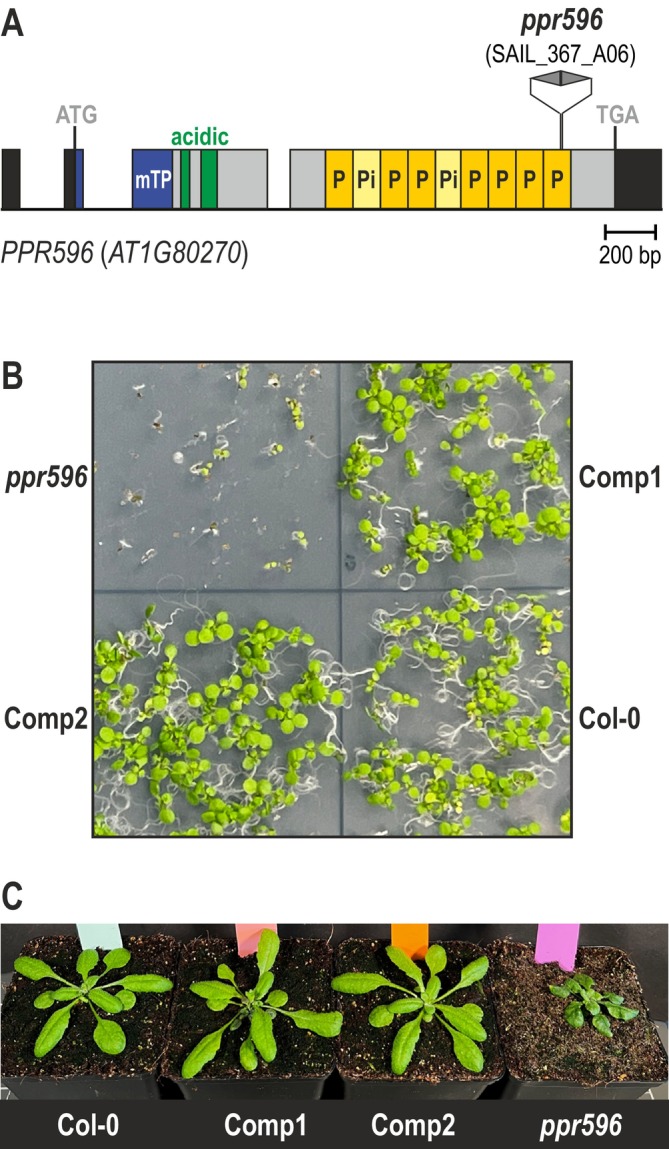
*PPR596* gene structure and growth of *ppr596* mutant and complemented lines. (A) Diagram of the *PPR596* gene located at locus AT1G80270, with exons represented as boxes and introns as solid lines according to the gene model available at www.TAIR.org (Reiser et al. 2024). Regions corresponding to untranslated regions and sequences coding for the mitochondrial transit peptide (mTP) as predicted by TargetP‐2.0 (https://services.healthtech.dtu.dk/services/TargetP‐2.0/ (Almagro Armenteros et al. 2019)) and PPR (P) and inferred PPR (Pi) motifs according to https://ppr.plantenergy.uwa.edu.au/ (Cheng et al. 2016; Gutmann et al. 2020) are indicated. The mature, processed protein has a predicted disordered domain with acidic stretches at its N‐terminus (https://www.uniprot.org/, (Uniprot 2025)). In the *ppr596* mutant line SAIL_367_A06, two T‐DNAs positioned as inverted repeats are inserted into the last PPR motif. (B) *ppr596* mutant and wild‐type (Col‐0) seedlings grown alongside two independent complemented lines (Comp1, Comp2) on half‐strength MS agar supplemented with 1% sucrose in a 16‐h photoperiod for nine days. (C) *ppr596* grown in a 16‐h photoperiod for five weeks and wild type (Col‐0) and two independent complemented lines (Comp1, Comp2) grown in a 16‐h photoperiod for three weeks are at similar developmental stages.

PPR596 is a mitochondrial protein (Doniwa et al. 2010) that, according to its array of P‐type and inferred PPR motifs (Figure 1A), sorts into the P subfamily. It is among the most abundant PPR proteins in Arabidopsis mitochondria (Fuchs et al. 2020). Members of the P subfamily have been described to contribute to correct transcript end maturation or splicing (Small et al. 2023). The Arabidopsis *ppr596* mutant used here for investigating molecular defects caused by PPR596 disruption has been previously studied (Doniwa et al. 2010). As reported by Doniwa and colleagues, the *ppr596* mutant germinated and developed with a major delay and displayed a twisted‐leaf phenotype (Figure 1B,C) described for various Arabidopsis mutants with major mitochondrial defects (Kühn et al. 2015, 2009; van Aken et al. 2010). We generated two independent complemented lines (Figure S1), for which re‐introduction of an intact *PPR596* gene copy restored plant growth to being wild type‐like (Figure 1B,C).

**FIGURE 2:**
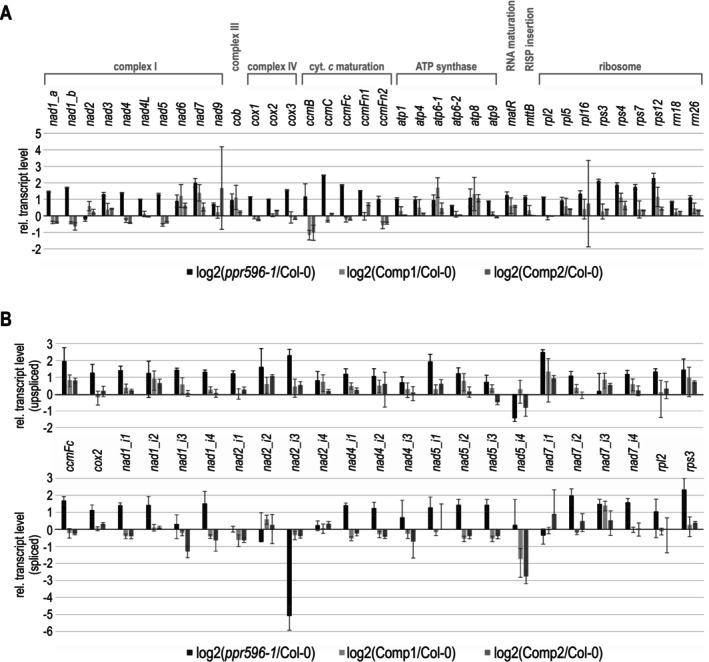
Inefficient *nad2* mRNA splicing in *ppr596*. (A) A quantitative RT‐PCR screen was performed to compare the abundances of mitochondrial transcripts between *ppr596*, its two complemented lines (Comp1, Comp2) and the wild type (Col‐0). Transcript levels for all protein‐coding and rRNA genes are depicted as log_2_ of the ratio of values determined in the mutant or complemented lines versus wild type for each transcript. Genes are arranged according to gene functions (RISP, Rieske iron–sulfur protein). Three technical replicates of two independent biological repeats were analyzed per genotype; error bars indicate standard deviations. The nuclear *18S rRNA* and *ACT* genes were used for data normalization. (B) Mitochondrial transcript splicing efficiencies were compared between the wild type (Col‐0), *ppr596*, and its two complemented lines (Comp1, Comp2) for all 23 mitochondrial introns, using a previously described RT‐qPCR assay (Falcon De Longevialle et al. 2007; Kühn et al. 2011). This assay quantifies unspliced transcripts by amplification of products that span exon‐intron junctions (upper diagram) and spliced transcripts by amplification of products that span splice junctions (lower diagram). The histogram depicts relative transcript levels as ratios compared with the wild type, using a log_2_ scale. Three technical replicates of two independent biological repeats were analysed per genotype; error bars indicate standard deviations. The nuclear *18S rRNA* and *ACT* genes were used for data normalization. Note that the primer pair used for *nad2* in the analysis shown under (A) amplifies across intron 2 and therefore cannot detect a specific decrease in intron 3 splicing.

To screen for potential mitochondrial gene expression defects in *ppr596*, mitochondrial transcript levels were compared between wild‐type, *ppr596*, and complemented seedlings (Figure 2A). For the nine intron‐containing mitochondrial genes, we additionally analyzed the accumulation of both spliced and unspliced mRNA species (Figure 2B). For *ppr596*, we found a pronounced decrease in *nad2* transcripts with excised intron 3. In the complemented lines, wild‐type‐like levels of *nad2* transcripts with spliced intron 3 were restored, showing that impaired excision of intron 3 from the *nad2* mRNA in the *ppr596* line was indeed due to PPR596 disruption. Apart from this distinct splicing defect and overall moderately increased mitochondrial transcript accumulation, no further major transcript changes were found in *ppr596*.

### 3.2 PPR596 Specifically Affects the Excision of the Third Intron of the *nad2* mRNA

**FIGURE 3:**
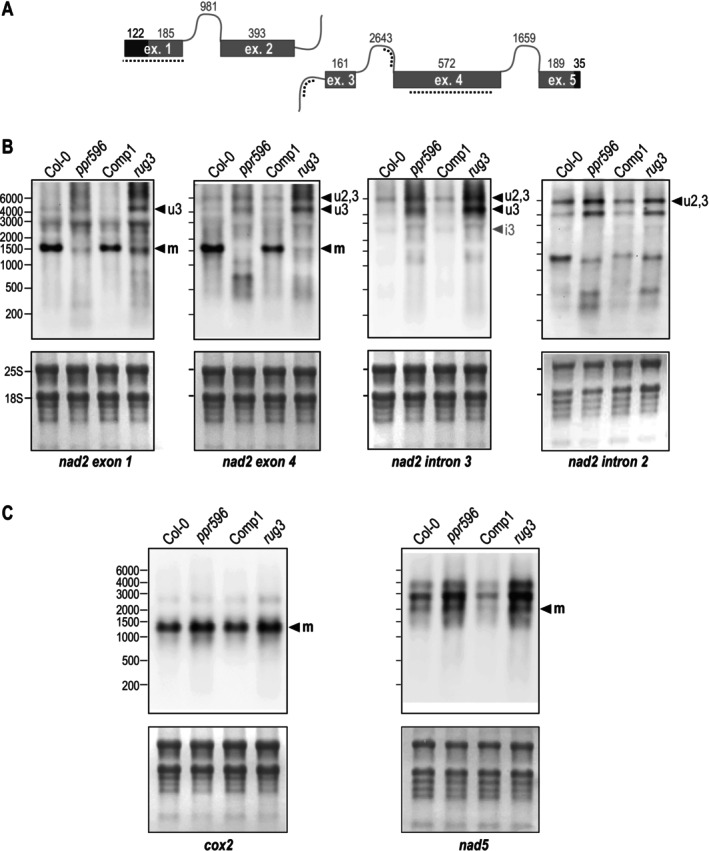
RNA gel blot hybridizations showing impaired splicing of *nad2* intron 3 in *ppr596*. (A) The *nad2* gene has five exons that are transcribed from two different mitochondrial genome regions and joined via trans‐splicing of intron 2. Lengths of exons and *cis*‐spliced introns are given in nucleotides according to (Forner et al. 2007; Sloan et al. 2018). Only exons are drawn to scale (grey, coding regions; black, untranslated regions); introns are depicted as grey lines. Dotted lines mark the target regions of probes used for RNA gel blot hybridizations. (B) Probes for exon 1, exon 2, intron 3, and intron 2 of the mitochondrial *nad2* mRNA were hybridized to filter‐immobilized total RNA isolated from seedlings of the wild type (Col‐0), *ppr596*, one complemented line (Comp1) and the *rug3* mutant used as a control (top panels). RNA size markers were run alongside samples; sizes are indicated in nucleotides. The same membranes stained with methylene blue are shown as a loading control in the lower panels. Signals corresponding to the mature mRNA (m), a pre‐mRNA from which intron 3 has not been excised (u3), and a pre‐mRNA containing intron 3 and the 3′ portion of intron 2 but no sequences co‐transcribed with exon 1 (u2,3) are indicated. A very faint signal (i3) detected in the wild type and the complemented line but not in the two mutants might correspond to the excised full‐length intron 3. (C) RNA gel blots were generated as described in (A) and hybridized using probes specific for *nad5* (exon 2) and *cox2* (exon 1). Signals corresponding to the mature mRNA (m) were assigned by inferring transcript sizes from annotated coding sequences (Sloan et al. 2018) and experimentally identified mRNA termini (Forner et al. 2007; Kühn et al. 2009, 2005).

The *nad2* coding sequence is disrupted by four introns, of which intron 2 is *trans*‐spliced and all other introns, including intron 3, are *cis*‐spliced (Figure 3A). Several mitochondrial proteins have been reported to specifically contribute to the efficient splicing of the third *nad2* intron in Arabidopsis, among them the PPR proteins ABO5 (Liu et al. 2010) and MISF26 (Wang et al. 2018), the RCC1‐family protein RUG3 (Kühn et al. 2011), and the mTERF family protein mTERF15 (Hsu et al. 2014). To validate the splicing defect found in *ppr596*, RNA gel blot hybridizations were performed, including a *rug3* mutant line next to *ppr596*, its complemented line, and the wild type (Figure 3B). Several probes were used for this analysis, each of them annealing to a different part of the *nad2* mRNA (Figure 3A). Probes specific for exon 1 or exon 4 yielded a distinct signal for the mature *nad2* mRNA in the wild type and complemented line. This signal was hardly detectable in *ppr59*6 or the *rug3* control. Both *ppr59*6 and *rug3* showed an overaccumulation of exon 1‐containing and exon 2‐containing transcripts larger than the mature *nad2* mRNAs. One of these transcripts had a size slightly above 4000 nt and was detected with probes against exon 1, exon 2, and intron 3 (signal labeled u3 in Figure 3B). Hence, this transcript corresponds to a *nad2* mRNA retaining intron 3, thus validating the importance of PPR596 for the efficient excision of this intron. Another transcript showing a slight overaccumulation in *ppr596* and *rug3* was detected with probes against exon 4, intron 2, and intron 3 at a size just below 6000 nt (signal marked u2,3 in Figure 3B). The slight increase seen for this pre‐mRNA in both mutants might be a consequence of the overall upregulation of mitochondrial transcript levels, which is a well‐documented secondary effect of mitochondrial dysfunction (Ayabe et al. 2023; Kühn et al. 2011; Sung et al. 2010), or of diminished efficiency of *nad2* intron 2 splicing. The former interpretation is supported by our RT‐qPCR analysis, which showed no decrease in mRNAs with excised intron 2 in *ppr596* (Figure 2B). In addition to *nad2*, RNA gel blot hybridizations inspected the accumulation of two further mitochondrial transcripts, *cox2* and *nad5* (Figure 3C). Like *nad2*, these mRNAs require group II‐intron excision but were not affected in splicing in *ppr596* according to our RT‐qPCR screen (Figure 2B). As expected, wild type‐like transcript patterns were seen in *ppr596* and *rug3* mutants for both *cox2* and *nad5*, with an overall increase in all detected transcript species that is attributable to secondary effects (see above). It has been pointed out that diminished levels of a spliced mRNA with concomitant elevation of its unspliced form are not sufficient to demonstrate a defect in splicing (Small et al. 2023). This is because a higher mRNA turnover, that is, upregulation of both transcription and RNA degradation, would lead to similar changes in spliced versus unspliced mRNA species. In the case of transcript changes seen in *ppr596*, this is an unlikely scenario because only one out of three introns on the same pre‐mRNA is affected in this manner.

Given that PPR596 disruption affects the excision of one particular intron, it is likely that PPR596 exerts its function on splicing by binding to a specific sequence of the *nad2* mRNA, hence promoting the folding of intron 3 into a splicing‐competent structure. Employing either the “PPR code” derived by (Barkan et al. 2012) or the expanded PPR motif nucleotide specificities derived by (Yan et al. 2019), we attempted to predict putative PPR596 targets within the sequence comprising exon 3, intron 3, and exon 4 of the *nad2* pre‐mRNA. These predictions failed to identify targets according to (Barkan et al. 2012) and detected 15 potential binding sites according to (Yan et al. 2019) that matched the expanded PPR code in only four out of nine positions (Figure S2). Therefore, the mode through which PPR596 could promote efficient *nad2* intron 3 excision cannot be reliably predicted. Several other nucleus‐encoded proteins have been shown to be required for the splicing of *nad2* intron 3 in Arabidopsis. In addition to ABO5, MISF26, RUG3, and mTERF15 that are highly specific for this intron (Hsu et al. 2014; Kühn et al. 2011; Liu et al. 2010; Wang et al. 2018), they include the RNA helicase ABO6 (He et al. 2012), the CRM‐domain proteins mCSF1 and CFM9 (Lee et al. 2019; Zmudjak et al. 2013), the zinc‐finger protein OZ2 (Bentolila et al. 2021) and the PORR‐domain protein RPD1 (Edris et al. 2024; Wang et al. 2024), all of which target several mitochondrial introns besides *nad2* intron 3. Considering the high number of factors that facilitate the splicing of this rather large intron, intron binding by PPR596 might also be influenced by the binding of other RNA‐binding proteins to this same intron, rather than by the intron sequence and folding only. Moreover, complexome profiling of Arabidopsis mitochondria detected PPR596 predominantly at sizes of 400–500 kDa (Rugen et al. 2021), implying its association with other proteins in vivo. It has been hypothesized that proteins required for efficient transcript splicing in mitochondria might be present in spliceosome‐like structures; however, the existence of such structures remains to be proven (Small et al. 2023). Regarding PPR596 binding sites, a thorough investigation of the evolution of both PPR596 and *nad2* intron 3 might contribute to narrowing them down. It should be noted that PPR proteins, though mostly acting as ssRNA‐binding proteins, can also associate with dsRNA helices (Waltz et al. 2020). Bearing in mind the large number of dsRNA domains in group‐II introns (Bonen and Vogel 2001), PPR596 modes of action other than ssRNA binding are possible.

### 3.3 Respiratory‐Chain Complex I Accumulation Depends on PPR596

**FIGURE 4:**
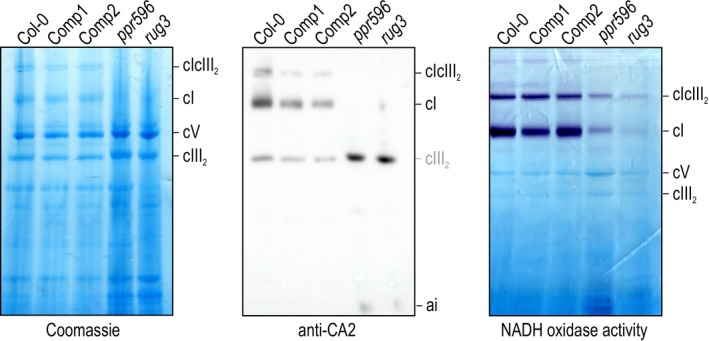
*ppr596* mitochondria have diminished levels of respiratory‐chain complex I. Mitochondria isolated from the wild type (Col‐0), two independent complemented lines (Comp1, Comp2), and mutant lines *ppr596* and *rug3* were solubilized using digitonin, and mitochondrial membrane complexes were separated by BN‐PAGE followed by Coomassie staining (left panel) or in‐gel activity staining for NADH oxidase activity (right panel). Bands corresponding to respiratory‐chain complexes I (cI) and III_2_ (cIII_2_), their supercomplex (cIcIII_2_), and to the ATP synthase (cV) are indicated. The middle panel shows BN‐PAGE‐resolved mitochondrial complexes probed with antibodies against the complex I subunit CA2. The signal marked ai corresponds to an early intermediate of complex I assembly that over‐accumulates in *ppr596* and *rug3* as a consequence of disruption of complex I assembly at the step of Nad2 insertion. Cytochrome *c*_*1*_, a subunit of complex III, has peroxidase activity and therefore reacts with the ECL reagent, leading to the production of a false‐positive signal at the level of cIII_2_.

Because of the major developmental delay shown by the *ppr596* mutant line, it was of interest to investigate potential consequences of impaired *nad2* mRNA splicing on mitochondrial biogenesis and function. We specifically focused on respiratory‐chain complex I, of which the Nad2 protein is a core subunit (Klusch et al. 2021). Blue‐native (BN)‐PAGE analysis of membrane complexes was performed on mitochondria isolated from the wild type, *ppr596*, two independent complemented lines, as well as the *rug3* mutant used as control (Figure 4). In this analysis, *ppr596*, like *rug3*, only differed from the wild type in having much less complex I and also less of the respiratory‐chain supercomplex composed of complex I and a complex III dimer. Accumulation of the complex III dimer alone and the ATP synthase was not affected. Consequently, the diminished supercomplex is caused by complex I not being available for supercomplex formation. To further validate the complex I defect resulting from impaired *nad2* mRNA splicing, complexes resolved by BN‐PAGE were immunolabeled with antibodies against the early‐assembling complex I subunit CA2 (Ligas et al. 2019) (Figure 4). This analysis detected complex I and the supercomplex in the wild type and complemented lines, but not in *ppr596*. *ppr596*, like *rug3*, instead showed overaccumulation of a low molecular weight complex containing CA2, which likely corresponds to the previously described 85‐kDa assembly intermediate of complex I (Ligas et al. 2019). This assembly intermediate is known to over‐accumulate if the immediately following assembly step involving Nad2 cannot proceed. Accordingly, complex I assembly in *ppr596* is impaired at the step of Nad2 insertion, which fits with defective *nad2* mRNA splicing in this mutant and adds to previous findings by Sayyed et al. (2024).

### 3.4 Minor Changes in *rps3* mRNA Editing Might Be Directly Related to PPR596 Disruption

The first reverse‐genetic study of PPR596 function had reported moderate changes in mitochondrial mRNA editing in *ppr596* (Doniwa et al. 2010). Editing of one specific site of the *rps3* mRNA corresponding to genome position 23,091 (numbering according to (Sloan et al. 2018)) was elevated in the mutant. It is unlikely that this change in editing contributes to the major developmental defect shown by *ppr596*. It is, however, possible that moderately altered editing in *ppr596* is a secondary effect of the impairment of *nad2* mRNA splicing and complex I biogenesis in this mutant. To see if defects in complex I can induce changes in editing, we looked at the editing of the *rps3* mRNA and of two additional, arbitrarily chosen mitochondrial transcripts, *cox2* and *cox3*, in *ppr596* and additional mutant lines. While we detected the same elevated editing at the previously described *rps3* site in *ppr596*, no such elevated editing was found in two other mutants defective in complex I, *ndufv1* (Kühn et al. 2015) and *rug3* (Figure S3). Hence, neither a complex I defect nor impaired *nad2* mRNA splicing induces altered *rps3* mRNA editing in the *ppr596* mutant. The change in *rps3* editing at position 23,091 in *ppr596* might accordingly be directly related to PPR596 disruption. None of the other tested sites was reproducibly altered in editing in all mutants when compared with the wild type and the complemented line (Comp1).

## 4 Conclusion

In summary, the work presented here identified PPR596 as a protein required for the efficient excision of the third intron from the *nad2* pre‐mRNA and, consequently, for the biogenesis of respiratory‐chain complex I. It adds yet another processing factor to the puzzling complexity of RNA metabolism in land plant mitochondria. In light of the reported high abundance of PPR596 (Fuchs et al. 2020), it is well possible that this protein has other roles besides promoting the splicing of one specific mitochondrial intron and directly or indirectly modulating *rps3* mRNA editing.

## Author Contributions

K.K. and E.H.M. planned research; L.P. performed most experimental work; V.C.W. performed preliminary experiments; L.P., K.K., T.S., and M.S.R. analyzed data; S.D., L.P., and E.H.M. generated resources; K.K. wrote the manuscript with input from all authors.

## Supporting information


**Data S1:** Supporting Information.

## Data Availability

The data that support the findings of this study are available from the corresponding author upon reasonable request.
