# Supplementary material for: PPR596 Is Required for nad2 mRNA Splicing and Complex I Biogenesis in Mitochondria of Arabidopsis thaliana
Source: Physiol Plant. 2025 Sep 9;177(5):e70507. doi: 10.1111/ppl.70507 (PMC12420532; doi:10.1111/ppl.70507)
Supplement: Supplementary file 1 — Data S1: Supporting Information. [file PPL-177-e70507-s001.pdf]

SUPPLEMENTARY INFORMATION

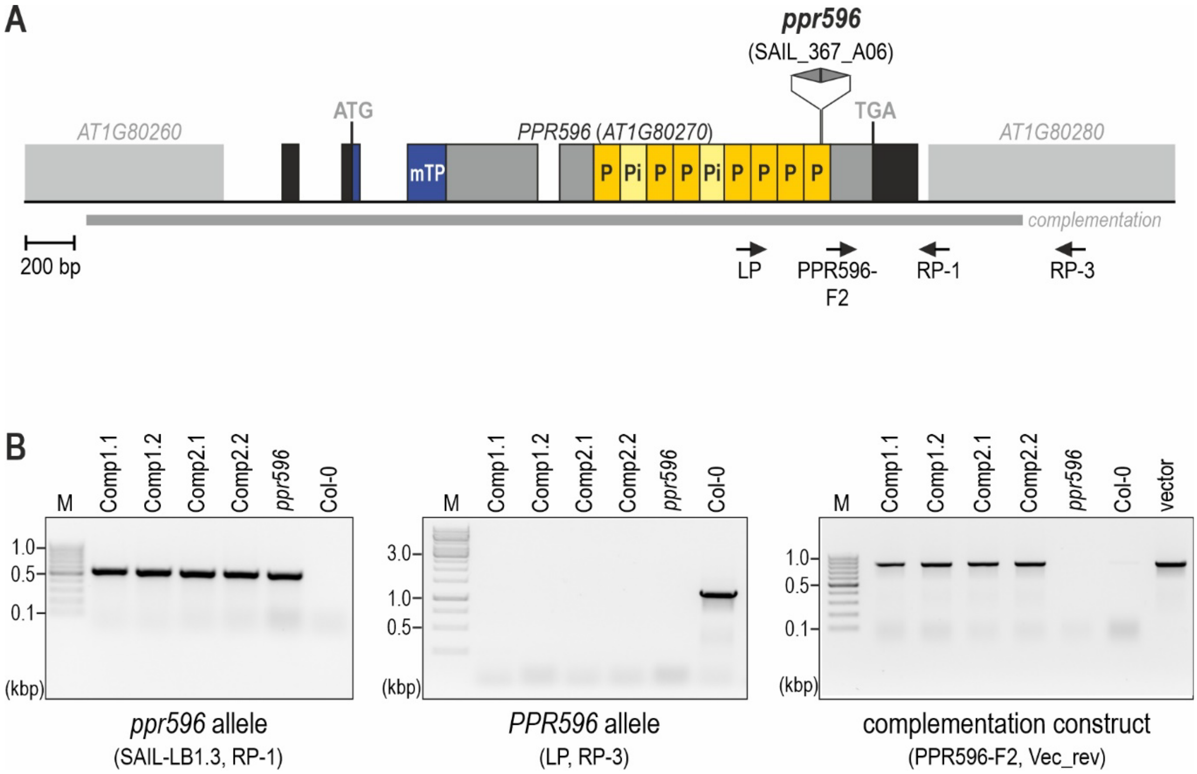

Supplementary Figure S1: Identification of complemented lines.

(A) Diagram of the AT1G80270 (*PPR596*) locus with its neighbouring loci; the region marked by a grey bar was re-introduced into the *ppr596* mutant for complementation. Note that exons and introns are only specified for AT1G80270. Annealing sites of gene-specific primers (arrows) used to verify the T-DNA insertion, the absence of a wild-type *PPR596* allele and the presence of the complementation construct are indicated. See Figure 1 for information on displayed *PPR596* gene features and Supplementary Table S1 for primer sequences.

(B) PCR analysis to verify the T-DNA insertion (*ppr596* allele), the absence of a wild-type *PPR596* allele and the presence of the complementation construct; primer combinations are indicated below gel images. Results are displayed for two individuals of each of the two complemented lines, Comp1 and Comp2. The *ppr596* mutant, the wild type (Col-0), and the T-DNA vector containing the complementation construct were used as controls.

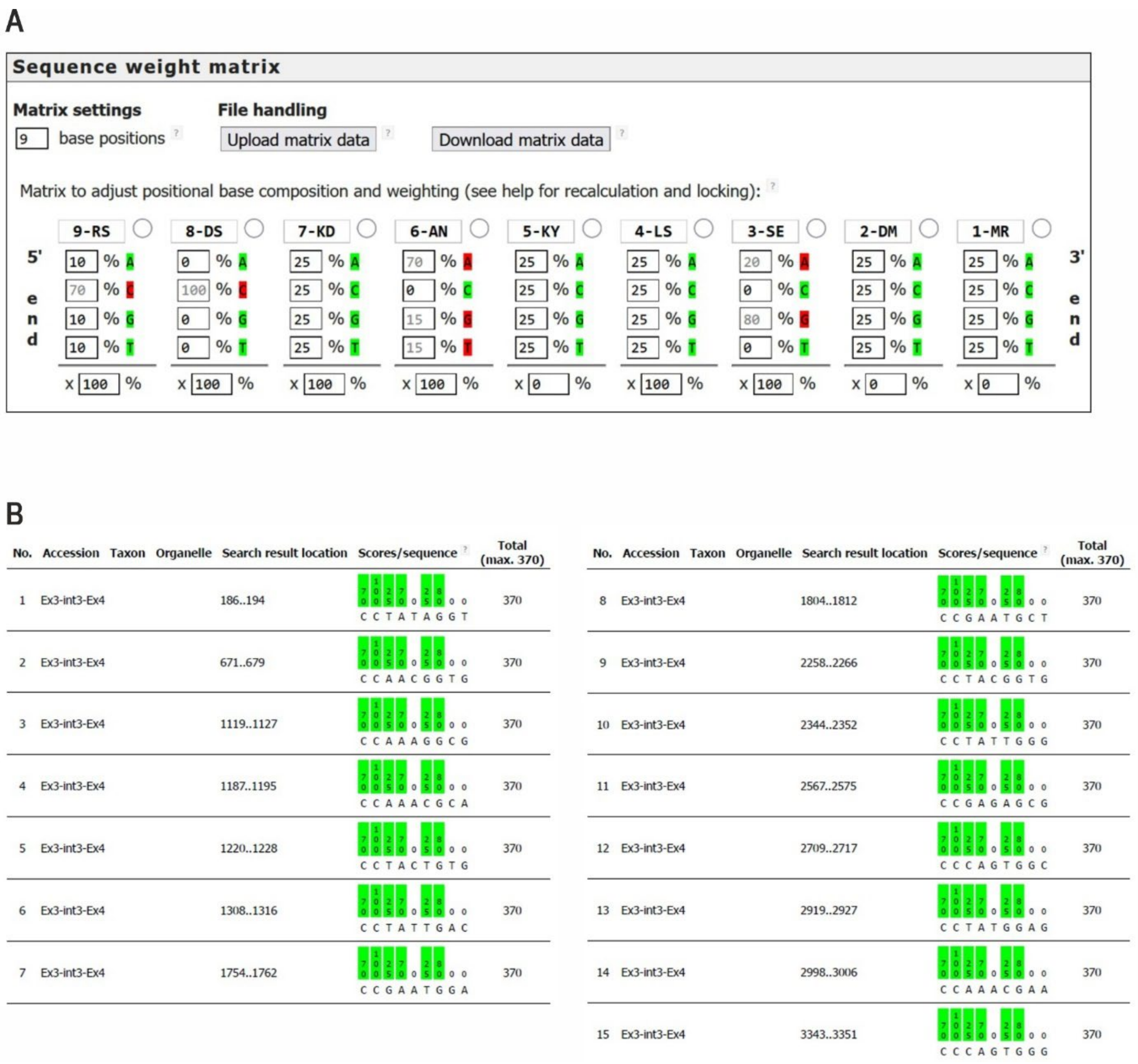

No.	Accession	Taxon	Organelle	Search result location	Scores/sequence	Total (max. 370)
1	Ex3-int3-Ex4			186..194	C C T A T A G G T	370
2	Ex3-int3-Ex4			671..679	C C A A C G G T G	370
3	Ex3-int3-Ex4			1119..1127	C C A A A G G C G	370
4	Ex3-int3-Ex4			1187..1195	C C A A A C G C A	370
5	Ex3-int3-Ex4			1220..1228	C C T A C T G T G	370
6	Ex3-int3-Ex4			1308..1316	C C T A T T G A C	370
7	Ex3-int3-Ex4			1754..1762	C C G A A T G G A	370
8	Ex3-int3-Ex4			1804..1812	C C G A A T G C T	370
9	Ex3-int3-Ex4			2258..2266	C C T A C G G T G	370
10	Ex3-int3-Ex4			2344..2352	C C T A T T G G G	370
11	Ex3-int3-Ex4			2567..2575	C C G A G A G C G	370
12	Ex3-int3-Ex4			2709..2717	C C C A G T G G C	370
13	Ex3-int3-Ex4			2919..2927	C C T A T G G A G	370
14	Ex3-int3-Ex4			2998..3006	C C A A A C G A A	370
15	Ex3-int3-Ex4			3343..3351	C C C A G T G G G	370

Supplementary Figure S2: Prediction of potential PPR596 binding sites.

(A) Weight matrix used for scanning the *nad2* exon3-intron3-exon4 region for putative PPR596 binding sites is shown. Each column corresponds to a nucleotide position and is labelled with the position of the PPR (counting from the C-terminus of the protein) and the specificity-defining fifth and last amino acid of the respective PPR. PPRs and fifth and last amino acids were extracted using the PPR finder sub-tool "PPR" (Cheng et al. 2016, https://ppr.plantenergy.uwa.edu.au/). None of the PPRs complied with the PPR-RNA binding code by Barkan et al. 2012. Nucleotide distributions were set to match the expanded PPR-RNA binding code derived by Yan et al. 2019, according to which four out of the nine PPR motifs showed an amino acid combination with a preference for particular nucleotides. Positional weights are shown below. No weight (0 %) was given to positions opposite to PPRs with amino acid combinations not tested for their binding capacities in Yan et al. 2019. Positions with 0 % do not have influence on target selection.

(B) Applying the weight matrix in (A) to the TargetScan tool of PREPACT (Lenz et al. 2018) identified 15 perfectly matching targets (max. score 370) in the region comprising Exon 3, Intron 3 and Exon 4 of *nad2* (3376 bp). Positions listed under “Search result location” refer to position 1 as the first nucleotide of Exon 3. Target no. 12 is found in the unusually large domain VI of intron 3.

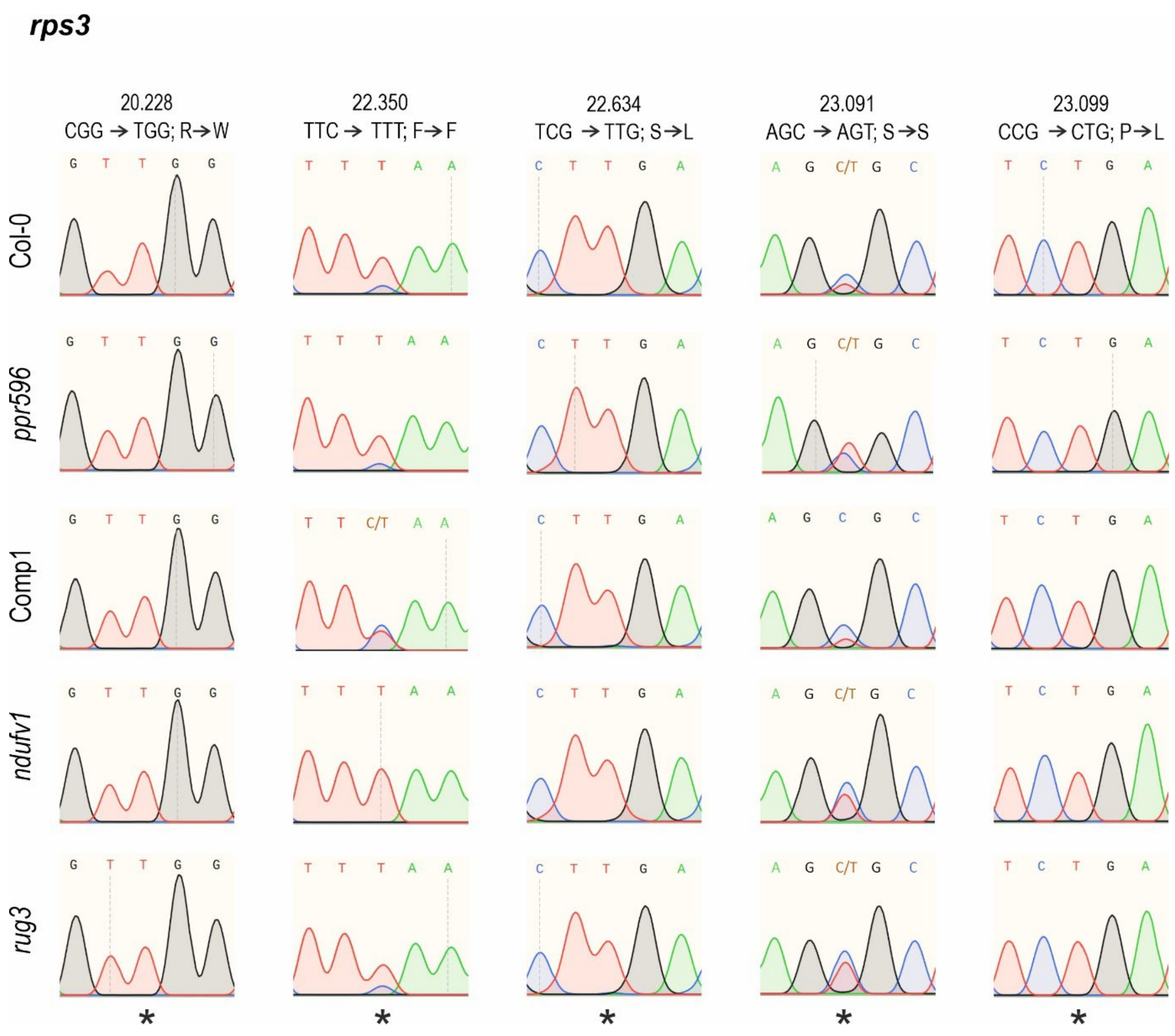

Supplementary Figure S3: Editing of *rps3*, *cox2*, and *cox3* mRNAs in the wild type (Col-0), *ppr596*, its complemented line Comp1, and mutants *rug3* and *ndufv1* compromised in complex I biogenesis. mRNA editing was assessed through cDNA sequencing. Editing sites, as annotated in the Arabidopsis Col-0 genome (accession NC_037304.1), are marked by an asterisk below the bottom row of chromatograms. Numbers given above each editing site refer to nucleotide positions in the Col-0 genome (Sloan et al., 2018).

rps3

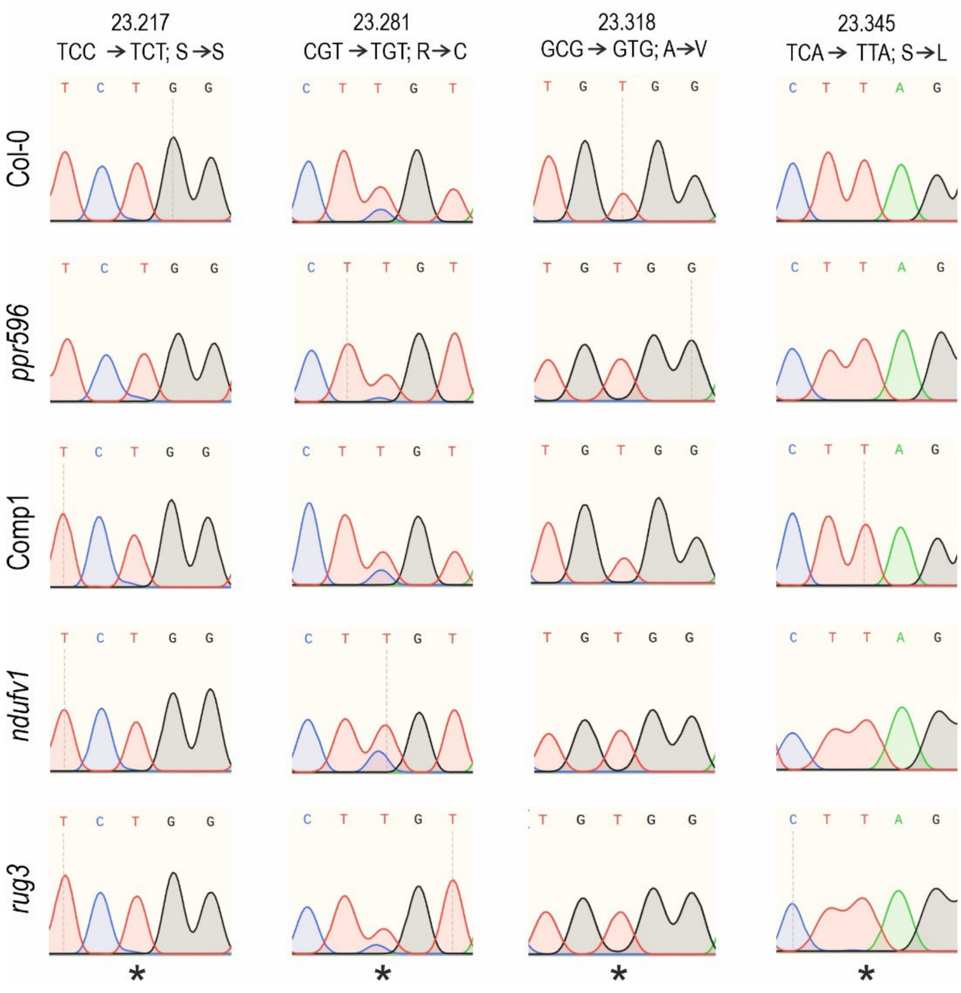

Supplementary Figure S3 - continued

cox2

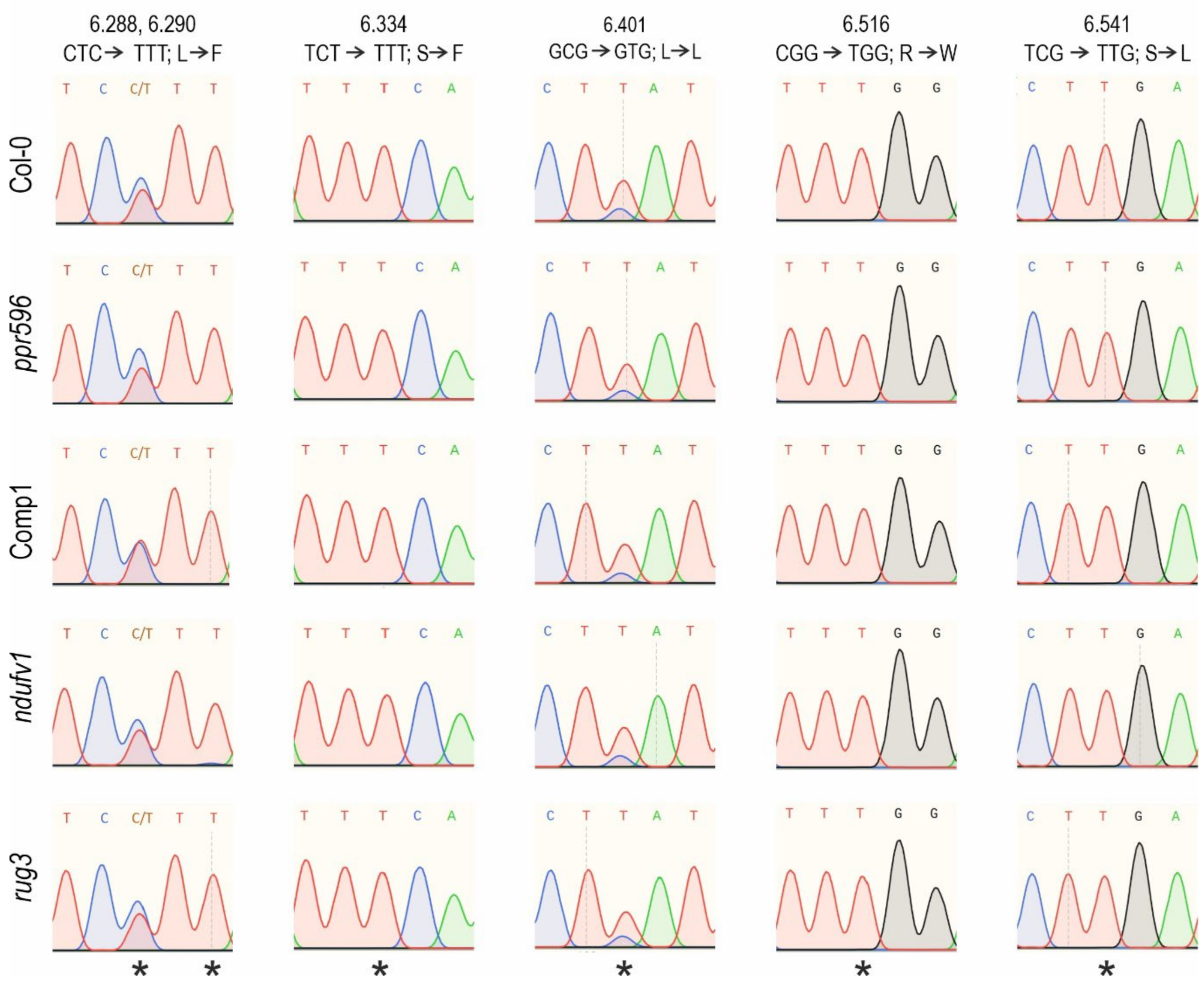

Supplementary Figure S3 - continued

cox2

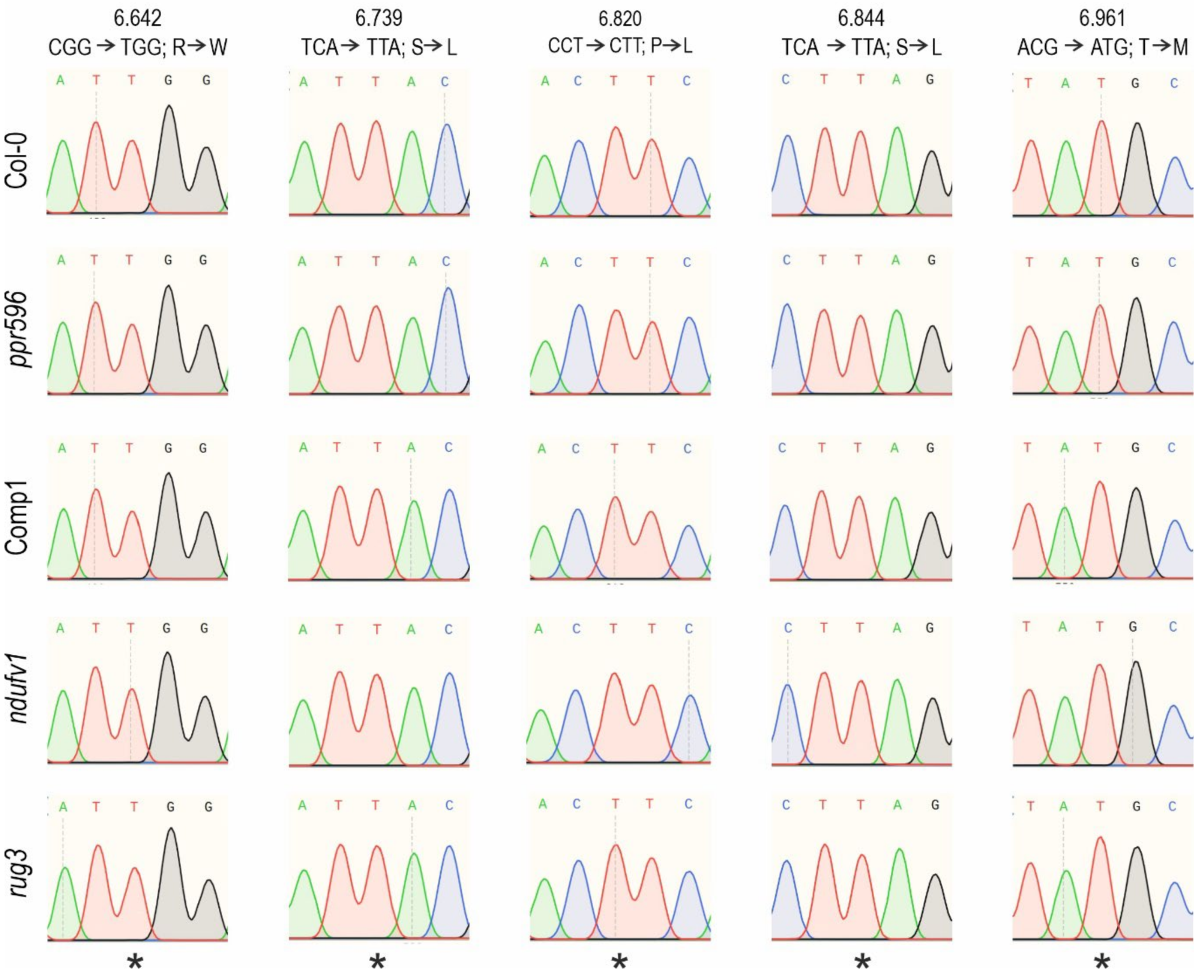

Supplementary Figure S3 - continued

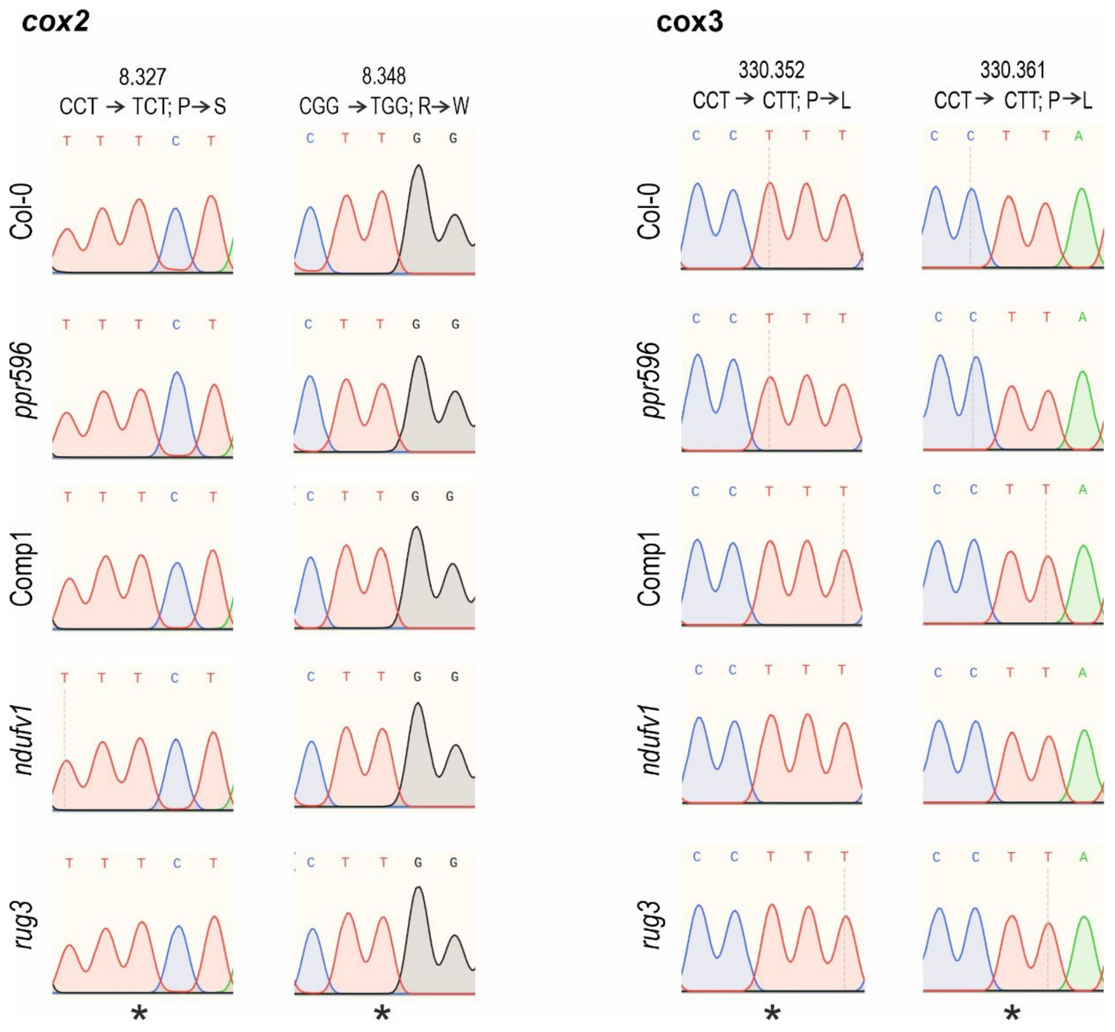

Supplementary Figure S3 - continued

cox3

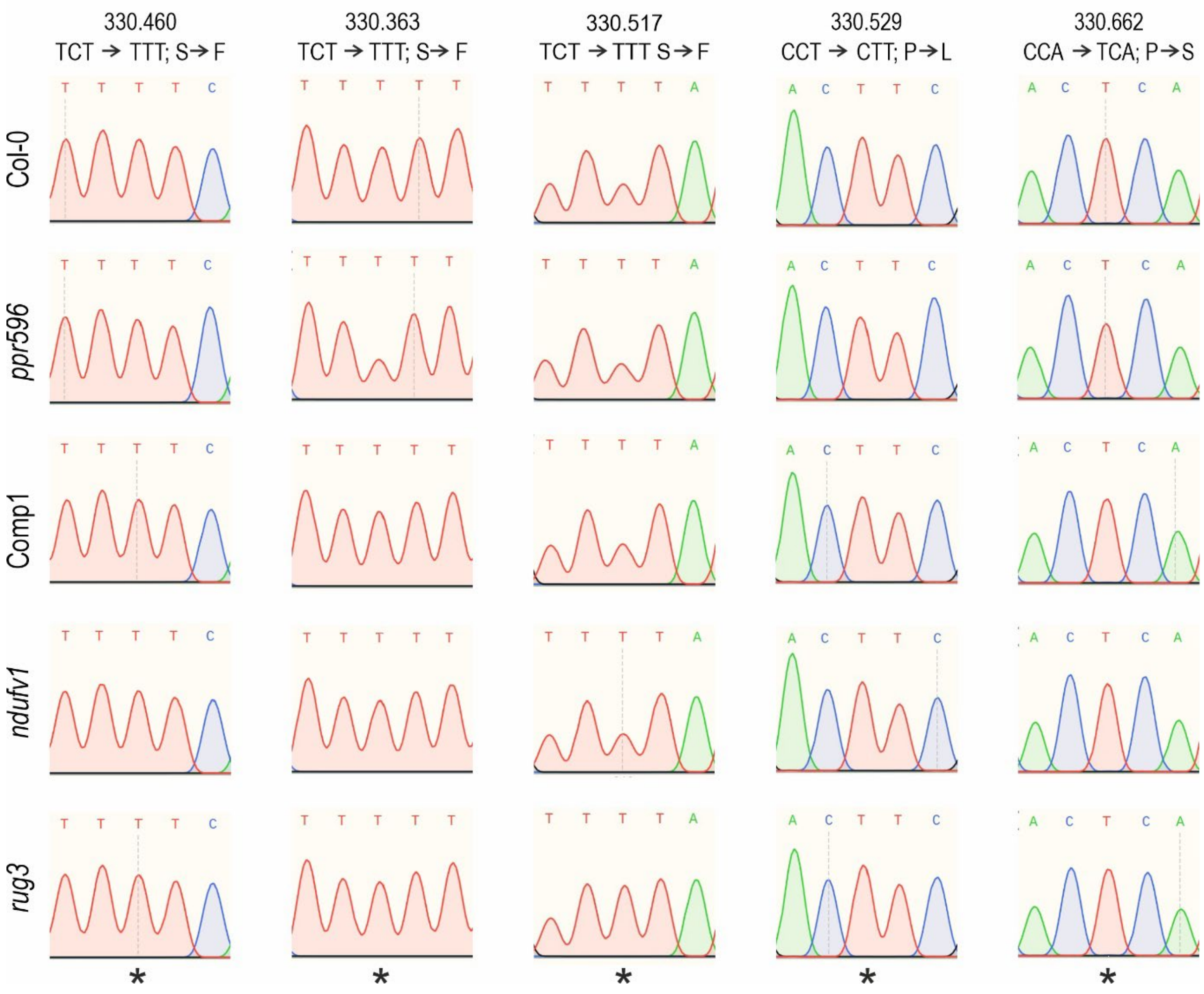

Supplementary Figure S3 - continued

Supplementary Table S1: Oligonucleotides used in this study

**Complementation construct primers**	**Sequence 5’-3’**
**PPR596_Comp_F**	GGCTAGATGCGAAATGGAAA
**PPR596_Comp_R**	ACATGGCTGCAAGAAGTGC
**Genotyping primers**	**Sequence 5’-3’**
**LP**	AAGCAGAGGCGATTTTTGAG
**RP-1**	TCAAACTGCGCGTATTCTACC
**Vec_rev**	TGCCCATTAACATCACCATCTAA
**RP-3**	GACTTGAGGGGAACAAGTGC
**PPR596-F2**	GAGATAGATTGAAGGCGGATAA
**SAIL_LB1.3**	GAATTTCATAACCAATCTCGATACAC
**Primers used for analysing transcript editing**	**Sequence 5’-3’**
**rps3_seqF**	GATCACTGAGGGGAAGGTTG
**rps3_seqF2**	GACCCCGTCGTAGTTCTCAA
**rps3_seqR1**	GCACCGAAGAATGGAAAGAG
**rps3_R2**	CGTTTCGGATATAGCACGTC
**cox2_F1**	ACCAGCCATTTCCGTCTTC
**cox2_R1**	CCCCTCCCTCACCTTACTCT
**cox2_seq1**	ATCCCGCAAAGGATTGTTC
**cox2_seq2**	GTGACTGCTCATCGGAACTG
**cox3_F1**	TCAATGCAATTAAAGAACCATCC
**cox3_R1**	ATCAAAGGGAGTGGGAAAGC

**Oligonucleotides used for generating probes for RNA gel blot hybridisation**		
**Probe name**	**primer name**	**Sequence 5’-3’**
***nad2* exon 1**	RPA-nad2e1-F1	CATTTTTTTATTGAGCCGAATCACT
	P3-nad2e1	TCCAAGCCAACCCACATTACTG
***nad2* exon 4**	nad2d-R	CCGGTTACAGCATTCCTTTC
	nad2d-F	AGCGGCGAAGAACAAATAGA
***nad2* intron 2**	nad2i2-N-F	AGTTAGAGAGGTTGGCGAACTACT
	nad2i2-N-R	AAAGTACCTCTCCAATCCTCGAT
***nad2* intron 3**	nad2i3-F	GTTCTGATAAGGAAGGAACAACCC
	nad2i3-R	TCTCTTTTCTAGTAGATGCCGAACC
***nad5* exon 2**	nad5-d011	TTACATCCATAAGTAGCTTGGTCCAT
	nad5-d012	AAGCGATGACCCTCTTTAGAT
cox2	cox2-d024	CTGTCAAAAGTGAGTGACTGCTC
	cox2-d023	GGCAATTAGGATCTCAAGACGCAGC

Primers used for quantitative RT-PCR		
**Transcript**	**Primer name**	**Sequence 5’-3’**
ACT	ACT-F	GGTAACATTGTGCTCAGTGGTGG
	ACT-R	AACGACCTTAATCTTCATGCTGC
nad5	nad5e4-F	AACTCGGATTCGGCAAGAA
	nad5e2-R	CTGGCTCTCGGGAGTCTCTT
nad9	nad9-F	GGATGACCCTCGAAACCATA
	nad9-R	CACGCATTCGTGTACAAACC
rpl16	rpl16-F	GAGCATTTGCCAAACTCACA
	rpl16-R	CGGACACTTTCATCGTGCTA
rps3	rps3-F	CCGATTTCGGTAAGACTTGG
	rps3-R	AGCCGAAGGTGAGTCTCGTA
cox2	cox2-F	TGATGCTGTACCTGGTCGTT
	cox2-R	TGGGGGATTAATTGATTGGA
ccmFc	ccmFc-F	CACATGGAGGAGTGTGCATC
	ccmFc-R	GTGGGTCCATGTAAATGATCG
rpl5	rpl5-F	AAGGGGTTCGACAGGAAAGT
	rpl5-R	CGTATTTCGACCGGAAAATC
cob	cob-F	TGCCGGAATGGTATTTCCTA
	cob-R	GCCAAAAGCAACCAAAACAT
nad6	nad6-F1	TCGTCGGAATACATCCTGTCT
	nad6-R1	TATGCCGGAAAGGTACGAAG
nad2	nad2e2-F	AATATTTGATCTTAGGTGCATTTTC
	nad2e3-R	AAAGGAACTGCAGTGATCTTGA
rps4	rps4-F	ACCCATCACAGAGATGCACA
	rps4-R	TCACACAAACCCTTCGATGA
atp6-1	atp6-1-F	TCTTTTGCGAGTCAATGCAC
	atp6-1-R	TCTCGCGTATCTCACATTGC
atp8	atp8-F	CCGTCGACTTATTGGGAAAA
	atp8-R	TTCCTTGGCCATGTACAACA
nad7	nad7-F	ACTGTCACTGCACAGCAAGC
	nad7-R	CATTGCACAATGATCCGAAG
nad1_b	nad1e4-F3	TGGGAGAGTATGCCAATATGA
	nad1e5-R2	GAAAATGGGAAGATCTAGGATAGG
nad4	nad4-F	AATACCCATGTTTCCCGAAG
	nad4-R	TGCTACCTCCAATTCCCTGT
atpB	atpB-F	GGATCAGCTTGCGAATTTGT
	atpB-R	GCAAATTGCTTCCCCACTAA
nad4L	nad4L-F	GGGGAATCCTCCTTAATAGACG
	nad4L-R	AACGAAAATGGCTAACCCAATA
cox3	cox3-F	CCGTAACTTGGGCTCATCAT
	cox3-R	AAACCATGAAAGCCTGTTGC

Transcript	Primer name	Sequence 5’-3’
ccmFn1	ccmFn1-F	AGCTCTTGGCATTGCTTTGT
	ccmFn1-R	AGTGCCACAATCCCATTCAT
ccmC	ccmC-F	AGCTACGCGCAAATTCTCAT
	ccmC-R	GCCGTGGCGATATAAACAAT
ccmFn2	ccmFn2-F	CGTGTCGTTCGTAATGGAAA
	ccmFn2-R	TGATAAGCCCACCAACTTCC
nad3	nad3-F	CGAATGTGGTTTCGATCCTT
	nad3-R	GCACCCCTTTTCCATTCATA
atp9	atp9-F	CATTCCCTCTGACGTCGAAT
	atp9-R	TCGTCGATTCTTACCCTCGT
nad1-a	nad1e1-F	TTGCCATATCTTCGCTAGGTG
	nad1e2-R	GACCAATAGATACTTCATAAGAGACCA
atp1	atp1-F	TCACTTCGACACGTCTTTGC
	atp1-R	GGAATGGCCTTGAATCTTGA
rps7	rps7-F	CTCGAACTGAACGCGATGTA
	rps7-R	AAGCTGCTTCAAGGATCCAA
cox1	cox1-F	GTAGCTGCGGTGAAGTAGGC
	cox1-R	CTGCCTGGATTCGGTATCAT
rrn18	rrn18-F	CGTCACCTGGGTCAAAAACT
	rrn18-R	GCTTGAAAACCGAAGTGAGC
rrn26	rrn26-F	GACGAGACTTTCGCCTTTTG
	rrn26-R	CTTGGAGCGAATTGGATGAT
ccmB	ccmB-F	TCTTGGAATCACATCCAGCA
	ccmB-R	CGAGACCGAAATTGGAAAAA
matR	matR-F	AATTTTTGCGAGAGCTGGAA
	matR-R	TTGAACCCCGTCCTGTAGAC
rpl2	rpl2-F	CCGAAGACGGATCAAGGTAA
	rpl2-R	CGCAATTCATCACCATTTTG
orfX	orfX-F	GGGGTCTTTCTTTGGAAACC
	orfX-R	TCTCCCTCATTCCACTCGTC
rps12	rpsl2-F	AGCCAAAGTACGGTTGAGCA
	rpsl2-R	TTTGGGTTTTTCTGCACCAT
atp6-2	atp6-2-F	TTCTGCCAGGAGTGCCTATT
	atp6-2-R	TTCTCGCTGGAGGTTCCTAA
n18	n18-F	AAACGGCTACCACATCCAAG
	n18-R	ACTCGAAAGAGCCCGGTATT
**rpl2_i**	323-rpl2i-F	TTAGGAAGAGCCGTACGAGG
	323-rpl2-R	CGCAATTCATCACCATTTTG
**rps3_i**	323-rps3-F	AGCCGAAAGGTGAGTCTCGTA
	323-rps3i-R	TCTACGGCGGGGTCACTAT
**cox2_i**	323-cox2-F	TGGGGGATTAATTGATTGGA
	323-cox2i-R	AGCAGTACGAGCTGAAAGGC

Transcript	Primer name	Sequence 5’-3’
**ccmFc_i**	323-ccmFc_i1-F	CCCGGATCGAATCAGAGTT
	323-ccmFc_e1-R	CACATGGAGGAGTGTGCATC
**nad4_i1 spliced**	323-nad4e1-F	ATTCTATGTTTTTCCCGAAAGC
	323-nad4e2-R	GAAAAACTGATATGCTGCCTTG
**nad4_i1 unspliced**	323-nad4i1-F	CCGTATGATGCGGAAGTCTC
	323-nad4e2-R	GAAAAACTGATATGCTGCCTTG
**nad4_i2 unspliced**	323-nad4i2-F	GCGGAACGACCAGAAAAATA
	323-nad4e3-R	TGCTACCTCCAATTCCCTGT
**nad4_i3 spliced**	323-nad4e3-F	TTCCTCCATAAATTCTCCGATT
	323-nad4e4-R	TGAAATTTGCCATGTTGCAC
**nad4_i3 unspliced**	323-nad4i3-F	TCTAGCTTGGTTCGGAGAGC
	323-nad4e4-R	TGAAATTTGCCATGTTGCAC
**nad5_i1 spliced**	323-nad5e2-F	TGGACCAAGCTACTTATGGATG
	323-nad5e1-R	CCATGGATCTCATCGGAAAT
**nad5_i1 unspliced**	323-nad5e2-F	TGGACCAAGCTACTTATGGATG
	323-nad5i1-R	TTCGCAAATAGGTCCGACT
**nad5_i2 unspliced**	323-nad5i2-F	GTACGATCGTGTCGGGTGA
	323-nad5e2-R	CTGGCTCTCGGGAGTCTCTT
**nad5_i3 unspliced**	323-nad5e4-F	AACTCGGATTCGGCAAGAA
	323-nad5i3-R	GCCGTGTAATAGGCGACCA
**nad5_i4 spliced**	323-nad5e5-F	AACATTGCAAAGGCATAATGA
	323-nad5e4-R	GTTCCTGCGTTTCGGATATG
**nad5_i4 unspliced**	323-nad5e5-F	AACATTGCAAAGGCATAATGA
	323-nad5i4-R	CCTGTAAACCCCCATGATGT
**nad7_i1 spliced**	323-nad7e1-F	ACCTCAACATCCTGCTGCTC
	323-nad7e2-R	AAGGTAAAGCTTGAAGATAAGTTTTGT
**nad7_i1 unspliced**	323-nad7i1-F	ACGGTTTTTAGGGGGATCTG
	323-nad7e2-R	AAGGTAAAGCTTGAAGATAAGTTTTGT
**nad7_i2 spliced**	323-nad7e2-F	GAGGGACTGAGAAATTAATAGAGTACA
	323-nad7e3-R	TGGTACCTCGCAATTCAAAA
**nad7_i2 unspliced**	323-nad7i2-F	AGTGGGAGAGCCGTGTTATG
	323-nad7e3-R	TGGTACCTCGCAATTCAAAA
**nad7_i3 unspliced**	323-nad7i3-F	TAAAGTGAAGTGGTGGGCCT
	323-nad7e4-R	CATTGCACAATGATCCGAAG
**nad7_i4 spliced**	323-nad7e4-F	GATCAAAGCCGATGATCGTAA
	323-nad7e5-R	AGGTGCTTCAACTGCGGTAT
**nad7_i4 unspliced**	323-nad7i4-F	CGGCCAAATGACTACAGGAT
	323-nad7e5-R	AGGTGCTTCAACTGCGGTAT
**nad2_i1 spliced**	323-nad2e1-F	GGATCCTCCCACACATGTTC
	323-nad2e2-R	GCGAGCAGAAGCAAGGTTAT
**nad2_i1 unspliced**	323-nad2i1-F	CCCATTCCTAACCAGTGGAG
	323-nad2e2-R	GCGAGCAGAAGCAAGGTTAT
**nad2_i2 unspliced**	323-nad2i2-F	TGTGGTGGTTGGGCCTAC
	323-nad2e3-R	AAAGGAACTGCAGTGATCTTGA

Transcript	Primer name	Sequence 5’-3’
nad2_i3 spliced	323-nad2e3-F	CTATGGGTCTACTGGAGCTACCC
	323-nad2e4-R	GCGCAATAGAAAGGAATGCT
nad2_i3 unspliced	323-nad2i3-F	GGCGAATTTCAAACTTGTGG
	323-nad2e4-R	GCGCAATAGAAAGGAATGCT
nad2_i4 spliced	323-nad2e4-F	TATTTGTTCTTCGCCGCTTT
	323-nad2e5-R	CAAAGGAGAGGGGTATAGCAA
nad2_i4 unspliced	323-nad2e4-F	TATTTGTTCTTCGCCGCTTT
	323-nad2i4-R	CTTATTCGTGGCAACCTTCC
nad1_i1 unspliced	323-nad1i1-F	CGTGCTCGTACGGTTCATAG
	323-nad1e2-R	GACCAATAGATACTTCATAAGAGACCA
nad1_i2 spliced	323-nad1e2-F	TCTGCAGCTCAAATGGTCTC
	323-nad1e3-R	ATTCAGCTTCCGCTTCTGG
nad1_i2 unspliced	323-nad1e2-F	TCTGCAGCTCAAATGGTCTC
	323-nad1i2-R	GGTTGGGTTAGGGGAACATC
nad1_i3 spliced	323-nad1e3-F	TCCGTTTGATCTCCCAGAAG
	323-nad1e4-R	AAAAGAGCAGACCCCATTGA
nad1_i3 unspliced	323-nad1i3-F	GGGAGCTGTATGAGCGGTAA
	323-nad1e4-R	AAAAGAGCAGACCCCATTGA
nad1_i4 unspliced	323-nad1i4-F	ACGGAGCTGCATCCCTACT
	323-nad1e5-R	AGCCCGGGATCTTCTTGA
